# The Medical Profession and the Medical Schools of New York

**Published:** 1851-08

**Authors:** 


*The Medical Profession and the Medical Schools of New York.* — The following article is from the New York Med. Gazette. Of the justness of the strictures on the profession of New York, we do not profess to judge. The article, however, contains significant truths which it may fairly be presumed are not restricted in their pertinency of application to any particular city; and for this reason we transfer it to our columns:

### THE MEDICAL PROFESSION AND THE MEDICAL SCHOOLS OF NEW YORK.

The question is so often discussed by the profession out of this city, Why does New York hold a secondary medical position as compared with Philadelphia, that it would, perhaps, be well if the profession and the professors in this city, should more frequently ask the same question. The fact can neither be gainsayed or denied. The first step towards improvement is to be fully aware of the necessity for such improvement. We do not, in this article, propose to answer the question by discussing the past medical history of New York; but only to call attention to what is palpably necessary to be done by our colleges and the profession at large, that New York may acquire her true position, as the medical metropolis of the country, as she already is and ever must remain the commercial metropolis. Its medical *advantages* and *resources* are more than double that of any other city in the Union, and if they were properly appreciated, brought out and made use of, they far exceed those of either Dublin or Edinburg.

If, indeed, the right men would come forward or could be pushed forward, so as to make available all the material which we have at hand in this city for a sound, thorough, practical medical education, New York would present higher claims on American medical students, and would offer more available advantages than Paris. For the study of the *elementary* principles of the profession in all its departments, Paris, for many years, has afforded opportunities exceeding that of any other city in the world, while the advanced student in either medicine, surgery, or obst trics, may pass his time more profitably in Dublin or Edinburg. This is because the right kind of men are at work there, who use all the material they have at hand for the advancement of the profession and their own scientific position. We are here very fond of *talking* about our great city and our unequalled advantages; but it is doubtful whether a large majority of the profession really know how great these advantages are. In our public hospitals during the last year, over *eighteen thousand* patients were treated, and if those who received medical aid from the dispensaries be added, the number will exceed *one hundred thousand.* Now out of this immense number of *public* patients there is afforded an abundant opportunity for the thorough study of any and every disease, medical and surgical, which is ever met in ordinary practice. With such a field for study and observation, any man of respectable talents, and the *requisite industry*, may become distinguished by his acquirements in any department of the profession he may select. The wonder is, that with such an open field there are so few laborers.

We have, to be sure, some among us who have acquired such a professional reputation as to do honor to the city. We can boast of a few names so distinguished as to have gained a world-wide fame; but the number is small in proportion to the size and natural advantages of this city, as compared with sister cities. There must be a large amount of talent undeveloped — of acquirement unknown and unproductive. Look at our Medical Journals for the last five years. The contributions from the Profession in New York are much less in number than those from either Philadelphia or Boston. We have but one society — the Pathological — which, so far as the medical public have any means of knowing, is a working society. The truth is, it seems to the Profession outside of this city as if the ultimate end and aim of New York medical men is to get patients, and get rich; and there are some reasons why this should be true. There is no other city in the Union where the practice of the Profession, as a trade, is so amply remunerated — none where the rewards or returns for time, labor, or money spent in the cause of science, is so small. There is no such thing as *esprit du corps* with the profession here, as a whole. It is limited to small and distinct circles — each one of which seems to regard itself as the natural enemy of all the rest.

There is no pride felt in our best and most distinguished men. On the contrary, they are the most prominent marks for abuse and detraction. Mediocrity alone is safe. Let a stranger come here and make inquiries in regard to any one of our most distinguished men, who has a conceded position outside of the sphere of conflicting interest or personal pique — be it a Mott or a Stevens, a Delafield or a Cheeseman, a Parker or a Green, a Clark or a Swett — and he probably will hear from seven of the first ten medical men that he may meet, only the most disparaging accounts. This is a peculiar characteristic of New York, and is a subject of frequent comment by the profession in other cities. It is this spirit that discourages all effort for high and honorable scientific distinction, except where it is sure of bringing its own reward, in securing practice. It is the fear of captious criticism and personal detraction which deters many from publishing, and thus contributing their quota to the general stock of professional knowledge.

In other cities, also, there is a pride and an interest felt in their medical schools by the profession. Here they are regarded with apathy or indifference, not to say with open hostility. The oldest school in the city is indebted very little to the fostering care and encouragement rendered by the profession; but to the talents, energy, and faithfulness of its Professors. The other schools must depend entirely for their success on the character which they establish by the efforts and talents of their respective Faculties.

The professional character of the city mainly rests upon its Schools, its Journals, and its Societies. Our three Medical Colleges now have a high mission to accomplish; and, fortunately, their prosperity depends upon the manner in which they do their duty. Medical students will inevitably go where they can obtain the best advantages, and be the best taught. If we in New York can offer the best lecturers, and can make it plain that we not only have far greater resources than any other city in the country, but that these resources are available for medical students, we shall soon have our full share. Each of the colleges will have of *bona fide* students all that they are entitled to. That college will be the most prosperous which displays the most energy and the most enterprise in advancing the interests of the students. The time has gone by when the reputation of a college rests upon the number of *names* on the Annual Catalogue. "*Dead heads*" add nothing to the character of a school or its funds. Enough of them would ruin any college.

We shall rejoice to see our schools stimulated by an honorable rivalry in the facilities they respectfully offer for acquiring a thorough medical education. We learn with pleasure that efforts are now being made in the right quarters to render clinical teaching in New York what it should be, and to make all of our hospitals schools for the practical study of disease.

